# NMR chemical shift assignment of UEV domain of ubiquitin-conjugating enzyme E2 variant 3 lactate dehydrogenase (UEVLD)

**DOI:** 10.1007/s12104-025-10240-7

**Published:** 2025-06-26

**Authors:** Jose G. Vazquez, David A. Nyenhuis, Marie-Paule Strub, Nico Tjandra

**Affiliations:** 1 Biochemistry and Biophysics Center, National Heart, Lung, and Blood Institute, National Institutes of Health, Bethesda, MD 20892 USA; 2 Protein Expression Facility, National Heart, Lung, and Blood Institute, National Institutes of Health, Bethesda, MD 20892 USA

**Keywords:** UEVLD, Tsg-101, AlphaFold

## Abstract

UEV domains are catalytically dead variants of the E2 enzymes which play an intermediate role in ubiquitin signaling. UEV domain containing proteins, like the ESCRT-I factor Tsg101 often play critical roles in trafficking of ubiquitylated cargos or in modulating ubiquitin processivity, or in determining the type of signal that is transferred to a target protein. Ubiquitin-conjugating enzyme E2 variant (UEV) and lactate/malate dehydrogenase (UEVLD), also known as UEV3, is a human paralogue of Tsg101 with apparent associations to cancer, innate immunity, NF-κB signaling, and autophagy. It contains an N-terminal UEV domain with 56% identity to that of Tsg101 and a C-terminal lactate dehydrogenase domain. Here, we show the backbone assignments of the UEV domain from UEVLD and find that its Cα shifts are consistent with a UEV domain composition. Further experiments suggest that it may have regions corresponding to the known binding pockets of Tsg101, but further structural and functional work will be required to uncover critical determinants of UEV domain function, and the role of these domains in Ubiquitin signaling as a whole.

## Biological context

Ubiquitin-conjugating enzyme E2 variant (UEV) and lactate/malate dehydrogenase (UEVLD), also known as UEV3, is a human paralogue of tumor susceptibility gene 101 (Tsg101) that was originally isolated from human placental cDNA (Kloor et al. 2002). Tsg-101 is a critical cellular factor that plays roles in trafficking and ubiquitin-mediated signaling. The N-terminal region of UEVLD harbors an apparent UEV domain with 56% identity to that of Tsg101 UEV, followed by a C-terminal lactate and malate dehydrogenase domain (Buljan et al. 2010). UEV domains are structurally homologous to canonical E2 enzymes, which constitute the intermediate step of ubiquitin signaling. They differ in the lack of a catalytic cysteine residue, which prevents the formation of the functional thioester bond with Ubiquitin. Still, UEV domains typically function in adjacent regulatory roles within the ubiquitin signaling framework.

In the case of Tsg101, the UEV domain is involved in both noncovalent Ub recognition and binding of P[T/S]AP motifs (Strickland et al. 2021). As a member of the endosomal sorting complex required for transport (ESCRT)-I, which plays a role in membrane remodeling events throughout the cell and is critical for endolysosomal trafficking, these binding events allow Tsg101 to recognize both ubiquitylated cargo and signal motifs present in other members of the ESCRT machinery (Strickland et al. 2021). Alternatively, the UEV domains of Ube2V1 and Ube2V2 form heterodimeric complexes with the E2 enzyme Ube2N, conferring K63 linkage specificity in NF-κB signaling and the DNA damage response, respectively (Zhao et al. 2018).

The biological function of UEVLD, and the role played by its UEV domain, is currently unknown. Still, UEVLD has been associated with reduced glucose consumption in tumor cells (Tu et al. 2021) and is dysregulated in glioblastoma and cervical cancers (Wang et al. 2023; Yang et al. 2023). UEVLD also has links to innate immunity, as it associates with cyclic AMP GMP synthase in the cGas/STING pathway (Lum et al. 2018) and to inflammation through identified associations with several members of the NF-κB signaling pathway (Van Quickelberghe et al. 2018), with the latter suggesting a connection to Ube2V1 and Ube2V2. Additionally, we have recently identified the ability of Tsg101 to chaperone certain E3 ligases, protecting them against degradation (Nyenhuis et al. 2025). UEVLD has identified associations with E3 ligases involved in NF-κB signaling (Traf2, Traf6) (Markson et al. 2009; Van Quickelberghe et al. 2018), autophagy (Parkin Sun et al. 2022; Namanja et al. 2019), neuronal development (Trim67) (Demirdizen et al. 2023) and the linear ubiquitin complex (SHARPIN) (Van Quickelberghe et al. 2018), implying that it may serve a similar role in modulating E3 stability.

Here, we present the backbone assignments of the UEV domain (residues 1–145) of UEVLD. Comparison of temperature dependence and R_2_ relaxation of the domain to the UEV domain from Tsg101 reveals similar hotspots to the Ub- and peptide-binding pockets of Tsg101. Additionally, comparison of Cα shifts to random coil values reveals that the domain likely adopts the expected UEV fold. This NMR assignment is a precursor for structural and biochemical studies to determine the function of UEVLD and to expand our knowledge of the determinants of UEV domain functionality as a whole.

## Methods and experiments

### Production and purification of isotopically enriched UEV domain from UEVLD

The ^15^N and ^13^C, ^15^N isotope labeled UEV domain from UEVLD (residues 1–145) with N-terminal His_6_ tag in pET-28B-vector was purified according to an existing protocol for the Tsg101 UEV domain (Strickland et al. 2017). Briefly, plasmid containing the UEVLD domain construct was transformed into Rosetta 2 (DE3) pLysS cells, and grown in minimal media (M9) supplemented with 2.0 g/L ^15^N ammonium chloride and 5.0 g/L ^13^C glucose (Cambridge Isotope) for isotopic enrichment. After induction with IPTG, expression proceeded overnight at 18 °C. Pellets were collected by centrifugation and solubilized in lysis buffer (100 mM Tris, 100 mM NaCl, 10% glycerol, pH 7.5) with added DNAse (benzonase nuclease, Millipore) and protease inhibitor (Roche). The resulting suspension was lysed with an Emulsiflex-C3 and pelleted by ultracentrifugation at 185,500×*g* for 60 min at 4 °C. The supernatant containing UEVLD was loaded onto a nickel column (HisTrap FF, GE Healthcare) equilibrated with the lysis buffer followed by gradient elution with the same buffer containing 0.5 M imidazole. The protein peak was collected and diluted into lysis buffer (four fold dilution), after which DTT and TEV protease were added for cleavage of the His6 tag overnight. Cleavage was checked by SDS-PAGE electrophoresis, and the tag was removed by a second run over the nickel affinity column, and the protein was spin-concentrated (Pall Macrosep, 3 k cutoff) prior to size exclusion chromatography using Superdex 75 column (GE Healthcare) that has been equilibrated with 20 mM KPi, 50 mM NaCl at pH 5.8 buffer. Finally, the protein was spin-concentrated into NMR buffer (20 mM KPi, 50 mM NaCl, pH 5.8) and stored at – 20 °C.

NMR data were acquired on either 600 MHz or 800 MHz Bruker NMR spectrometers equipped with cryogenic probes and at 25 °C, unless otherwise specified. Samples were prepared with 300 µL of protein in NMR buffer and 20 µL of D_2_O in a Shigemi tube. Protein concentrations were checked via A280 using the extinction coefficients of the UEV domains of UEVLD (22,920 M^−1^ cm^−1^) and of Tsg-101 (25,900 M^−1^ cm^−1^), with final protein concentrations of 400 μM for triple resonance assignment experiments, 200 μM for dynamics experiments, and 100 μM for temperature dependence. For backbone assignment, triple resonance HNCO, CBCA(CO)NH (Grzesiek and Bax 1992), and HNCACB (Wittekind and Mueller 1993) were collected at 600 MHz. Additionally, a 3D ^15^N-edited NOESY-HSQC with mixing time of 90 ms was collected at 800 MHz. [^1^H-^15^N]-HSQC experiments were collected at both 600 and 800 MHz.

The ^15^N transverse relaxation was determined at 800 MHz for both the UEV domains of UEVLD and Tsg101 from an experiment with the following relaxation delays: 8, 16, 32, 48, 64, 80, 96, 112, and 128 ms. Intensities were extracted for each peak at a given delay, and the resulting intensity series was fit to a single exponential decay using the PRISM software.

For comparison of the temperature dependence between Tsg101 UEV and the UEV domain of UEVLD, CSPs were estimated according to the equation (Strickland. et al. 2017):

$$CSP_{HN} = \sqrt {\left[ {0.5(\left( {H_{{33\,\, ^{ \circ } {\text{C}} - 7 ^{ \circ } {\text{C}}}} } \right)^{2} + \left( {\alpha \left( {N_{{33 ^{ \circ } {\text{C}} - 7 ^{ \circ } {\text{C}}}} } \right)} \right)^{2} } \right)} ,$$

where the scaling factor α for nitrogen is equal to 0.14 and CSPs were calculated as the difference in the shift observed at 33 °C and 7 °C for each protein. All temperatures were calibrated using and referenced to TSP.

All NMR spectra were initially processed using NMRPipe (Delalgio 1995) and CcpNMR AnalysisAssign v3.1 (Skinner et al. 2016) where further processing was done using python and GraphPad PRISM.

### Extent of assignments and data deposition

**Fig. 1:**
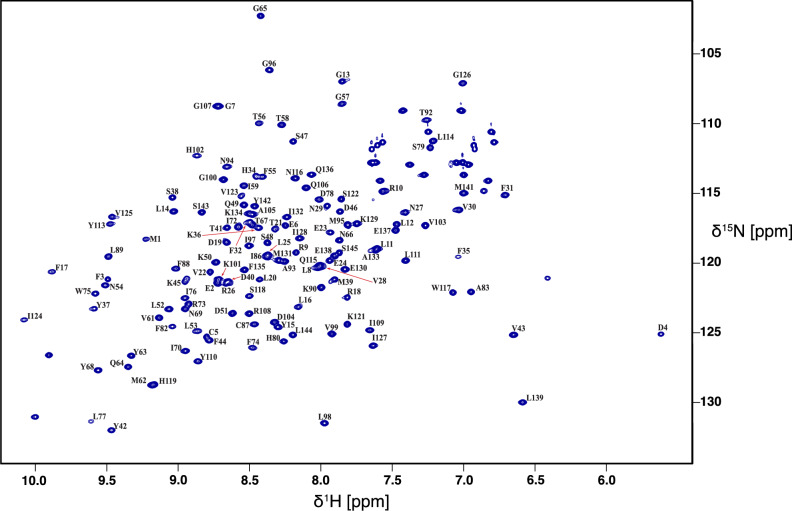
The backbone assignments of the UEV domain of UEVLD (residues 1–145). The 2D [^1^H-^15^N]-HSQC spectrum of the UEV domain of UEVLD was obtained at 800 MHz proton frequency and 25 °C. Peaks are labeled with their corresponding assignments

Backbone resonance assignments were determined for the UEV domain (residues 1–145) of UEVLD. All expected amide resonances (accounting for ten prolines) were assignable excepting the N-terminus. The Cα resonances were assigned for all residues except Pro84 and Cβ resonances were assigned for all except Ser47 and Pro84. Chemical shift results for apo UEVLD are available under BMRB accession code: 53013 and the [^1^H-^15^N]-HSQC spectrum is shown in Fig. 1.

### Structural comparisons

**Fig. 2:**
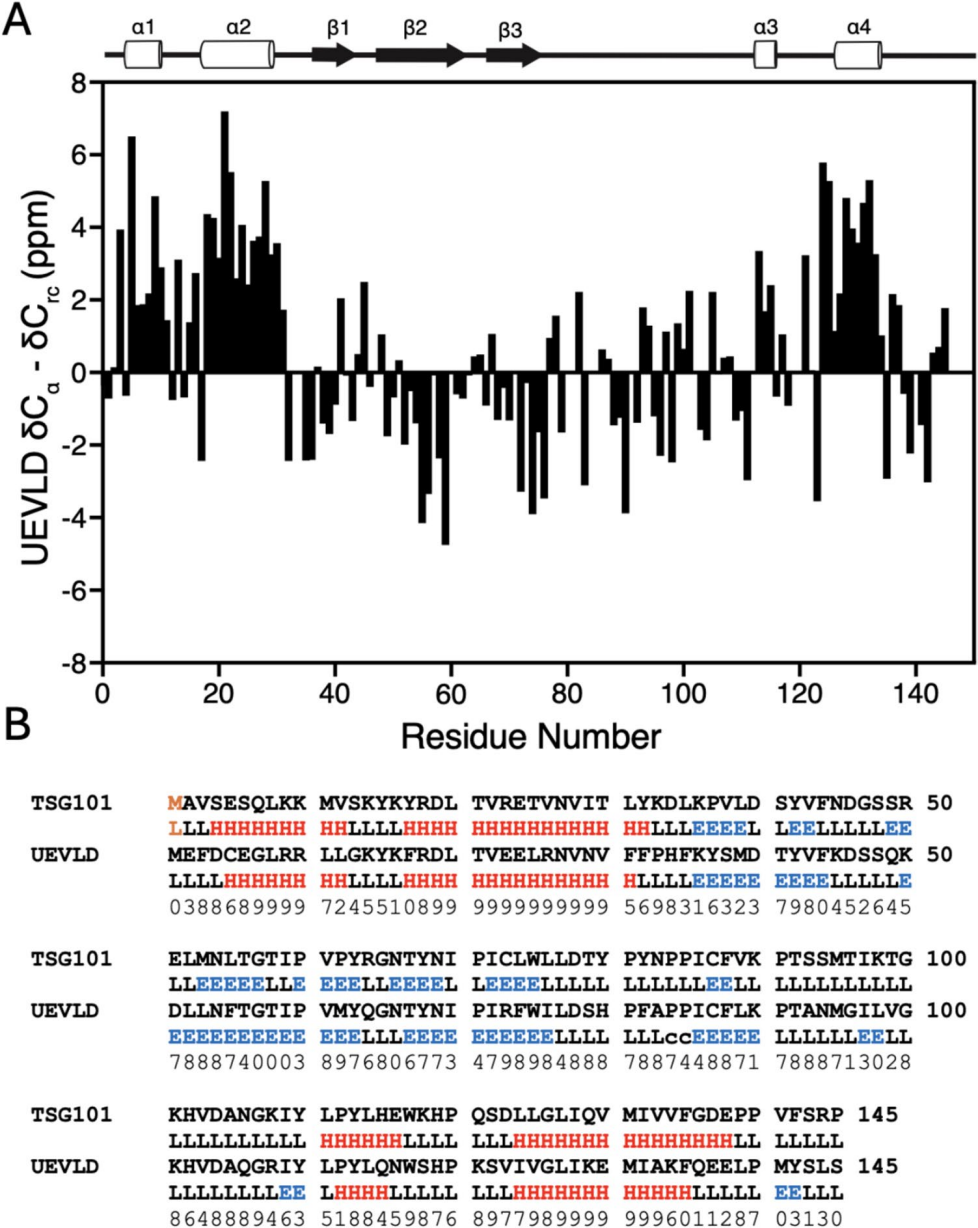
**A** Secondary Cα carbon chemical shifts are consistent with the expected E2 fold for the UEV domain of UEVLD. The secondary shifts for the Cα carbon (Spera and Bax 1991; Wishart et al. 1995) of the UEV domain were plotted for each position. Contiguous positive and negative Cα secondary shift regions are consistent with the expected four helices and four ß-strands that comprise a typical E2 secondary structure composition and are present in the UEV domain of Tsg101 (Pornillos et al. 2002) .**B** comparison of secondary structure in the UEV domain of Tsg101 (Top, PDB ID: 1KPP state 1) to that predicted for the UEV domain of UEVLD using the software package Talos-N with backbone HN, N, Cα, and Cß shifts (Shen and Bax 2013). The prediction confidence is given from the Talos-N output, and in both cases helices are colored red, and ß-sheets blue. As with the Cα plot, the Talos-N prediction shows the same four helices and three sheets for UEVLD as in Tsg101. The UEVLD prediction shows slightly more ß-character, but these regions are typically one to two residues long with low confidence scores. Note that to align the sequences, an N-terminal methionine (orange) was inserted into the Tsg101 sequence which is not present in the 1KPP structure. Secondary structure codes for the first state of 1KPP were output from Pymol

The Tsg101 UEV domain displays a typical “E2 fold” signature consisting of four helices adjacent to a four-stranded β-sheet (Pornillos et al. 2002). The Cα shifts from the UEV domain of UEVLD were thus compared to random coil values (Spera and Bax 1991; Wishart et al. 1995) to look for similar structural elements (Fig. 2A). Positive Cα secondary shifts potentially corresponding to four alpha helices are visible. In Tsg101, helices two and four are longer, and this is consistent with the data for UEVLD. Additionally, three regions with negative secondary Cα shift are observed, consistent with β-strands, and a fourth strand may exist around residue 90, as in Tsg101. Secondary structure prediction with Talos-N using the UEVLD amide, Cα, and Cß shifts (Fig. 2B) also suggests four helices and three sheets coincident with those of Tsg101 (taken from the first state of PDB ID: 1KPP) (Shen and Bax 2013). There is slightly more predicted ß-character in UEVLD, but these are one or two residue stretches with generally low confidence. Overall, the assignments are consistent with the “E2-type” fold seen for Tsg101 UEV.

**Fig. 3:**
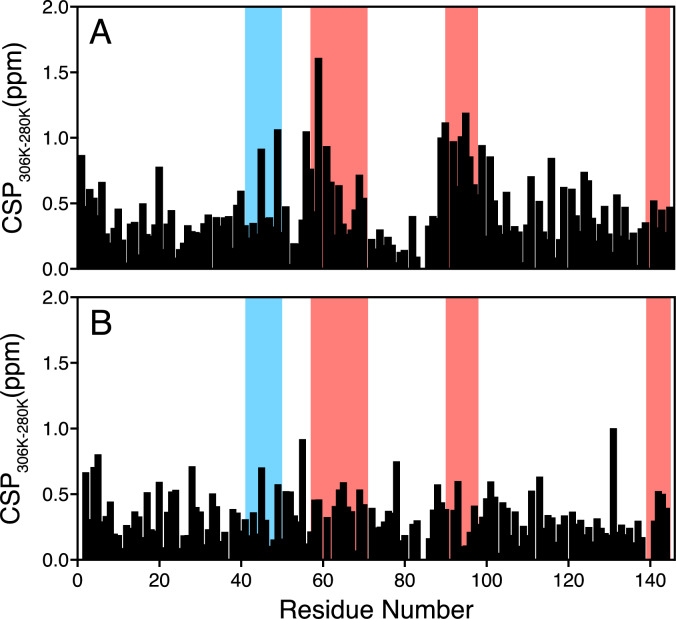
Chemical shift perturbations comparing the temperature response of the UEV domains of UEVLD (**A**) in comparison to that of Tsg-101 (**B**). CSPs were calculated as the difference between the chemical shift at 33 °C and 7 °C for each domain. UEVLD displays heightened differences in several regions, which map to residues that comprise the P(T/S)AP pocket of Tsg101 (red) and part of the Ub-binding pocket involving an extended ß-hairpin (blue). These regions were determined from the NMR structure of Tsg101 in complex with HIV-1 PTAP (PDB ID: 1M4Q) and the Xray structure of Tsg101 in complex with Ub (PDB ID: 1S1Q) using pymol, as being within 4 Å of the given binding partner. For UEVLD, they were determined by structural alignment to an Alphafold 3 prediction of the UEV domain of UEVLD (Abramson et al. 2024)

**Fig. 4:**
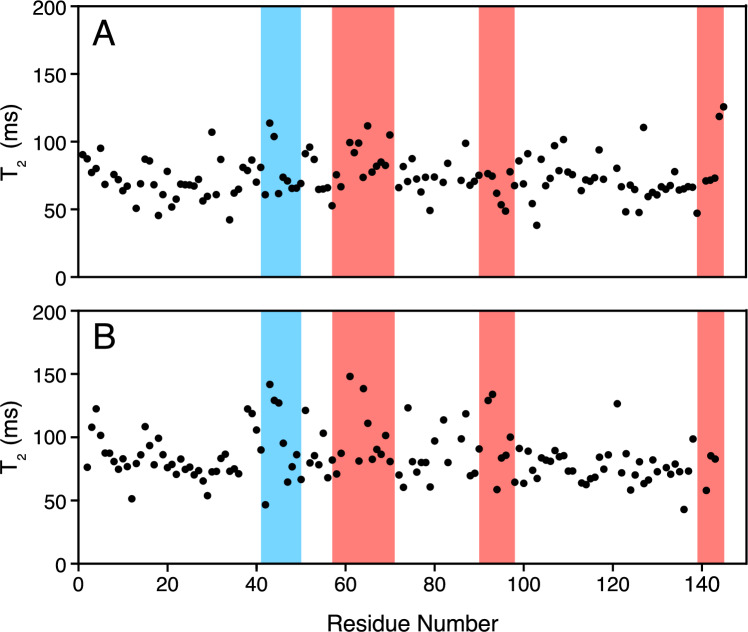
^15^N transverse relaxation times of UEVLD (**A**) and Tsg-101 (**B**) plotted against the residue number of the respective protein. The.^15^N transverse relaxation times were acquired at 800 MHz and 25˚C. The P(T/S)AP pocket (red) and UB binding ß-hairpin region (blue) are colored as in Fig. 2. Both proteins have moderate variation in relaxation times across the protein, with heightened differences in the two known binding pockets of Tsg101

The Tsg101 UEV domain harbors two known binding pockets, which bind Ubiquitin and P(T/S)AP peptides, respectively. We next looked at the temperature dependence of chemical shifts for the UEV domains of Tsg101 and UEVLD, as well as their ^15^N transverse relaxation times, to look for similarities that might be hallmarks of these regions in UEVLD. Plotting the temperature effect as chemical shift perturbations (CSPs) observed between 33 ℃ and 7 °C for both proteins (Fig. 3), shows that there are much larger shifts in the UEV domain of UEVLD than in Tsg101. Interestingly, the largest shifts are seen in regions corresponding to the peptide (red) and Ub (blue) binding pockets in the Tsg101 UEV domain. Looking at the transverse relaxation times of both proteins at 25 °C (Fig. 4) reveals that Tsg101 actually undergoes more fluctuations under these conditions, but that both proteins have increased fluctuations around these apparent binding sites. These results suggest that in addition to the E2 fold typical of UEV domains, UEVLD may harbor Ub and peptide binding pockets, although further study will be required to determine if they function similarly to those of Tsg101.

## Discussion

Here, we show the backbone assignments for the N-terminal UEV domain of UEVLD, an understudied paralogue of the ESCRT-I factor Tsg101. The obtained Cα shifts relative to random coil values are consistent with the expected UEV fold, as in Tsg101, and ^15^N transverse relaxation experiments and temperature dependence suggest that the protein may have regions analogous to the Ub and P(T/S)AP binding pockets in Tsg101, which would warrant further study. Tsg101 is an essential cellular factor involved with other elements of the ESCRT machinery in membrane remodeling processes and endolysosomal trafficking, while other UEV domain containing proteins, such as Ube2V1 and Ube2V2, function in concert with E2 enzymes to facilitate or alter ubiquitin signaling (Strickland et al. 2021; Zhao et al. 2018). The functional role of UEVLD is still uncertain, but its known associations with hallmarks of cancer, and to proteins involved in innate immunity, NF-κB signaling, and autophagy suggest that, like the other UEV domains it may be involved in a regulatory capacity adjacent to the Ubiquitin signaling machinery (Markson et al. 2009; Sun et al. 2022; Van Quickelberghe et al. 2018). Future work will be required to tease apart the presence of UB, peptide binding, or other functional surfaces on the UEV domain of UEVLD, and to contextualize these elements into the proteins broader cellular function, with the potential to also learn more about the unique determinants that have driven the formation of these catalytically “dead” E2 enzyme variants. Ultimately, the assignment of the NMR signals of the UEV region of UEVLD will provide the necessary foundation to determine the three-dimensional structure for future functional characterization and identification of key functional determinants relative to Tsg101.

## Data Availability

No datasets were generated or analysed during the current study.
